# Circadian transcriptome processing and analysis: a workflow for muscle stem cells

**DOI:** 10.1002/2211-5463.13629

**Published:** 2023-05-20

**Authors:** Valentina Sica, Oleg Deryagin, Jacob G. Smith, Pura Muñoz‐Canoves

**Affiliations:** ^1^ Department of Medicine and Life Sciences Universitat Pompeu Fabra Barcelona Spain; ^2^ Altos labs Inc San Diego CA USA

**Keywords:** bioinformatics, circadian rhythms, muscle stem cells, satellite cells

## Abstract

Circadian rhythms coordinate biological processes with Earth's 24‐h daily light/dark cycle. In the last years, efforts in the field of chronobiology have sought to understand the ways in which the circadian clock controls transcription across tissues and cells. This has been supported by the development of different bioinformatic approaches that allow the identification of 24‐h oscillating transcripts. This workflow aims to describe how to isolate muscle stem cells for RNA sequencing analysis from a typical circadian experiment and introduces bioinformatic tools suitable for the analysis of circadian transcriptomes.

## Graphical abstract

Muscle stem cells, ‘satellite cells’, replenish muscle fibers upon damage, and their function is influenced by circadian clocks. Characterizing circadian rhythms in satellite cells has been challenging in this low abundance cell type with available methodologies. Here, we share a workflow to compare oscillating transcripts in mouse satellite cells starting from cell isolation and ending with bioinformatic analyses.

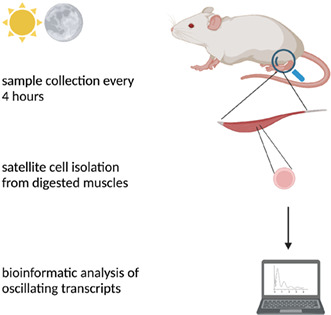

**Abbreviations**
BH: Benjamini–Hochberg
BICW: Bayesian information criterion weight
BMAL1: brain and muscle ARNT‐like 1
CLOCK: circadian locomotor output cycles kaput
CRY: cryptochrome
DESeq2: differential gene expression analysis
DODR: detection of differential rhythmicity
DryR: differential rhythmicity analysis in R
FACS: fluorescence‐activated cell sorting
FDR: false discovery rate
GC: guanine‐cytosine
GOBP: gene ontology biological processes
GSEA: gene set enrichment analysis
JTK: Jonckheere–Terpstra–Kendall
KEGG: Kyoto Encyclopedia of Genes and Genomes
LimoRhyde: linear models for rhythmicity, design
MsigDB: molecular signatures database
Nr1d1: nuclear receptor subfamily 1 group D member 1
Nr1d2: nuclear receptor subfamily 1 group D member 2
PCA: principal component analysis
PER: period
PSEA: phase set enrichment analysis
RAIN: rhythmicity analysis incorporating nonparametric methods
ROR: retinoic acid receptor (RAR)‐related orphan receptor
Sva: surrogate variable analysis
TPM: transcript per million
ZT: Zeitgeber time

Animals, plants, fungi, and bacteria have all evolved circadian timing mechanisms to align and adapt physiological processes and behaviors in response to daily environmental cues. Circadian, from the Latin ‘circa diem’ (‘about a day’), refers to a period of roughly 24 h, the time that Earth takes to perform a single rotation on its axis. Virtually every cell within the body possesses a molecular circadian clock that is capable of self‐sustaining oscillations yet can adapt in response to exogenous zeitgebers (time givers), such as light, time of food intake, and exercise. At the molecular level, oscillations of circadian genes are regulated by a complex feedback loop controlled by a core set of transcription factors and their regulators. The essential regulator in mammals is BMAL1 (brain and muscle ARNT‐like 1), which dimerizes with CLOCK (circadian locomotor output cycles kaput) and binds to E‐box elements to activate the transcription of controlled clock genes (CCGs). Among the CCGs regulated by the BMAL1/CLOCK complex are *period* (*Per*) and *cryptochrome* (*Cry*), whose encoded proteins also dimerize and act as inhibitors of BMAL1/CLOCK transcriptional activity. Proteasomal degradation of PER and CRY subsequently releases the repression of BMAL1/CLOCK. A series of auxiliary loops also exist, including nuclear receptor subfamily 1 group D member 1 (Nr1d1 or REV‐ERB‐α) and member 2 (Nr1d2 or REV‐ERBβ), which inhibit BMAL1 transcription, and retinoic acid receptor (RAR)‐related orphan receptor (ROR), which conversely activates BMAL1 transcription [1]. The transcriptional effects of BMAL1 regulation help to drive the 24‐h rhythms of transcription of between 10% and 30% of genes in the genome, depending on the tissue [2]. Indeed, a key feature of circadian rhythms is that the circadian transcriptional program, that is, the 24‐h oscillating genes, can vary greatly from one tissue to another due, in part, to the contribution of cell type–specific transcription and epigenetic regulators. Recent findings also reveal that communication both between tissues [3, 4] and within a tissue type [5, 6] is critical in defining the circadian transcriptional program within each organ [7]. For tissues in the periphery, such communication may also include local clock‐independent effects, driven by systemic effects from SCN‐generated behavior cycles such as feeding‐fasting [3, 6, 8]. These conceptual advances have been supported by bioinformatic analyses that have classified rhythmic genes in each tissue.

## Bioinformatic tools to define circadian transcriptomes

Since the late 1970s, a variety of methods for rhythmic gene detection have been developed and evaluated by chronobiology researchers, including parametric methods, such as Lomb‐Scargle [9] and ARSER [10], as well as numerous nonparametric methods, of which the Jonckheere‐Terpstra‐Kendall algorithm for rhythmic gene detection (JTK_CYCLE) [11] is most widely used. These important tools have been invaluable in driving the field, and the bioinformatic tools are currently evolving to better capture circadian programs in a quantitative manner. Each tool has its own limitations. For example, the JTK_CYCLE detection scope is limited to symmetric cosine waveforms and excludes any asymmetric rhythms, such as sawtooth‐shaped. As such, it will not capture the full profile of day/night differences in a given dataset, and this limitation should be considered accordingly. However, a number of other nonparametric algorithms, such as RAIN [12] (Rhythmicity Analysis Incorporating Nonparametric methods) and empirical‐JTK_CYCLE‐with‐asymmetry [13], have been developed to fill this gap by detecting both symmetric and asymmetric waveforms. Among the recently developed methods, BioCycle utilizes a deep neural network trained with both real‐world and synthetic data, which enables the detection of symmetric, asymmetric, periodic, and aperiodic waveforms [14].

Another challenge the field has become aware of in recent years concerns difficulties in determining whether the rhythmicity of a particular gene is different between two or more conditions. For example, it is now well established that the classically used ‘Venn diagram’ approach (overlapping 24‐h oscillating genes identified using algorithmic detection performed separately for two or more groups, often compared regardless of their rhythmic phase) can lead to an overestimation of differences [15]. In parallel, phase set enrichment analysis (PSEA) [16] can be used for phase‐specific enrichment of gene sets for each group and, thus, for further comparison of rhythmic processes and their phase alignment between the groups. However, the definition of rhythmic processes for PSEA within individual groups has the same weaknesses as the above algorithms for the definition of rhythmic genes, and only the most significant gene sets can be implicitly trusted. In response to such limitations, specific algorithms for differential rhythmicity analyses have been developed that focus on differences in rhythmic expression based on linear regression with rhythmic category classification into the amplitude change (gain or loss of rhythmicity), phase change, or unaltered rhythms [15]. Earlier methods in this direction, such as DODR [17] (Detection Of Differential Rhythmicity) and LimoRhyde [18], test the hypothesis of whether two rhythms are statistically different, while CircaCompare [19] provides a means to quantify differences specific to the desired rhythmic characteristic (mesor, amplitude, and phase). However, prior to hypothesis testing, a set of prefiltered rhythmic transcripts is defined using nonparametric (e.g., JTK_CYCLE or RAIN) or parametric (e.g., limma [20]) methods. By contrast, model selection frameworks, such as dryR [21] and compareRhythms [15], do not require prior rhythmic transcriptome definition and fit different linear models for each rhythmic category (including arrhythmic genes), with a subsequent choice of the best model based on an information‐theoretic criterion. Interestingly, besides the model selection approach, compareRhythms also allows various traditional tools to be tried for hypothesis testing, while dryR allows multiple groups to be compared at a time and to include more than one categorical co‐variate for batch correction. It must be noted that the problem of arbitrary thresholds remains to an extent, despite advancements in novel differential rhythmicity analysis methods. As such, each method must also be validated by manual inspection of data (see Tips below).

## Circadian rhythms in stem cells

One understudied aspect of circadian regulation is the role of the circadian clock in stem cells. For instance, the circadian transcriptome of muscle stem cells (satellite cells) is subject to extensive rewiring during aging, which interestingly can be rescued in part through caloric restriction [22, 23]. However, the role that circadian clocks themselves play in satellite cells is poorly understood. To facilitate such research, we have recently optimized a protocol and workflow (from isolation to bioinformatic analyses) for determining the rhythmic transcriptome in this technically challenging cell type.

## Materials

GentleMACS Octo Dissociator (Miltenyi Biotec, Bergisch Gladbach, Germany, 130‐095‐937).

Dulbecco's Modified Eagle's medium (DMEM) containing liberase (Roche, Basel, Switzerland #177246).

Fetal bovine serum (FBS) (Sigma‐Aldrich, Gillingham, UK, F7524).

Liberase (Roche, 5401020001).

Dispase (Gibco, #17105‐041).

Calcium chloride (CaCl_2_) (Thermo Scientific, Waltham, MA, US, J63122).

Magnesium chloride (MgCl_2_) (Invitrogen, Carlsbad, CA, US, 2672831).

Penicillin–streptomycin (P‐S) (Sigma‐Aldrich P4458).

RBC lysis buffer 1× (eBioscience, 00‐4333‐57).

40‐, 70‐μm and 1‐μm cell strainer (Clearline 141378C, 141379C, 141380C).

PE‐Cy7‐conjugated anti‐CD45 (Biolegend, San Diego, CA, US, 103114).

anti‐Sca‐1 (Biolegend, 108114).

PE‐Cy7‐conjugated anti‐CD31 (Biolegend, 102418).

Alexa Fluor 647‐conjugated anti‐CD34 (BD Pharmigen, San Diego, CA, US 560230).

PE‐conjugated anti‐α7‐integrin (AbLab, Vancouver, Canada, 53‐0010‐05).

RNeasy micro kit (Venlo, The Netherlands, Qiagen‐cat. no./ID: 74004).

## Methods

### Sample preparation: from harvesting muscles to muscle stem cell isolation

To generate a circadian transcriptome, samples are collected at equal intervals over the circadian cycle (for full guidelines on guidelines for circadian experimental setups, see Hughes et al. [24]) Typically, a minimum of 6‐time points is collected with a biological number of replicates of 3 or more. Special care must be taken to account for sex differences, as these have been reported to exert a significant effect on the identity of circadian output genes [25].

To isolate satellite cells from mice:

1. Euthanize mice by cervical dislocation and excise skeletal muscles. Dissect muscles from fore and hind limbs (abdominal muscle can also be included if necessary; gluteus and diaphragm are not usually collected) and place them in a 50 mL Falcon tube containing about 20 mL of DMEM plus 1% Penicillin–streptomycin (P‐S), preferably placed on ice.
2. Harvest and mince muscles manually.
3. Minced muscles (1 g) are enzymatically digested at 37 °C for 1 h, using the gentleMACS Octo Dissociator (Miltenyi Biotec, 130‐095‐937). Digestion buffer is composed of DMEM media containing 1% P‐S, liberase (0.1 mg·g^−1^ muscle weight) (Roche, 5401020001; 5 mg·mL^−1^), 0.3% dispase (Gibco, #17105‐041), 0.5 μm CaCl_2_, and 6 μm MgCl_2_.
4. After digestion, immediately place the tubes on ice.
5. Add DMEM, 1% P‐S, and 10% FBS (Sigma‐Aldrich F7524) to dilute the digestion buffer and stop the enzymatic reaction.
6. Filter once through a 100‐μm cell strainer and once through a 70‐μm cell strainer.

Mice were bred at the animal facility of the Barcelona Biomedical Research Park, housed in standard cages under 12‐h light–dark cycles, and fed *ad libitum* with a standard chow diet. The Catalan Government approved the work protocols, following applicable legislation.

#### Tip 1

To ensure a rapid and clean filter step, gently centrifuge (2 min 50 ***g*** 4 °C) to separate the remnants of tissues. Filter first the supernatant and then the resuspended pellet (in 10 mL of the DMEM, 1% P‐S, and 10% FBS).

7 Centrifuge 10 min 670 g 4 °C.
8 Discard the supernatant.
9 Resuspend the pellet in RBC lysis buffer 1× (eBioscience, 00‐4333‐57), for 5 min on ice, protected from light.
10 Add cold PBS until 30 mL and filter over a 40‐μm cell strainer.
11 Centrifuge 10 min 670 g, 4 °C.
12 Resuspend the pellet in FACS (fluorescence‐activated cell sorting) buffer (PBS, 2.5% FBS) and count the cells.
13 Centrifuge 670 g 4 °C and resuspend the cells in FACS buffer (100 μL/1 × 10^6^ cells) containing 1 : 200 PE‐Cy7‐conjugated anti‐CD45 (Biolegend, 103114), 1 : 200 anti‐Sca‐1 (Biolegend, 108114), 1 : 200 PE‐Cy7‐conjugated anti‐CD31 (Biolegend, 102418) (used for lineage‐negative selection), 1 : 50 Alexa Fluor 647‐conjugated anti‐CD34 (BD Pharmigen, 560230), and 1 : 200 PE‐conjugated anti‐α7‐integrin (AbLab, 53‐0010‐05) (used for double‐positive staining of quiescent satellite cells), for 30 min on ice, protected from light.

#### Tip 2

Cells can be frozen after antibody incubation by adding FACS buffer, centrifuging 10 min at 670 g 4 °C, and covering the pellet in a freezing medium (FBS/10% DMSO).

14 Once the incubation with antibodies is completed, add FACS buffer up to 30 mL, centrifuge 10 min 670 g 4 °C, and then resuspend the cell pellet in 450 mL of FACS buffer for satellite cell sorting.

#### Tip 3

The number of satellite cells sorted per mouse can vary (from about 50 000 to 200 000 cells), depending on the mouse model and age. The number of cells required can be sorted directly in the RLT buffer for RNA extraction, taking into account the efficiency of the buffer.

#### Tip 4

The day of the RNA extraction can produce a batch effect to be corrected in the bioinformatic analyses. If possible, it is recommended to perform the RNA extraction on the same day or randomize the samples in order to reduce the introduction of biases.

### RNA extraction and quality control

Given the paucity of satellite cells, they can be first sorted via fluorescence‐activated cell sorting (FACS), and RNA can then be extracted using an RNeasy micro kit (Qiagen‐cat. no./ID: 74004) following the manufacturer's instructions. It is highly recommended to perform the DNase incubation step, even though it is suggested as an optional step in the protocol. Clean RNA is essential for the following assessments. After RNA extraction, RNA quality and concentration are analyzed using a 2100 Bioanalyzer or a Fragment Analyzer and an Agilent RNA 6000 pico kit suitable for RNA of an estimated concentration of 50–50 000 pg·mL^−1^. To be suitable for sequencing, the RNA should have a RIN (RNA Integrity Number) of at least 7/10 and an rRNA Ratio (28S/18S) of 2.

### Low‐input RNA sequencing

An aliquot of total RNA (0.3 ng) is used to generate barcoded RNA‐seq libraries using the NEBNext Single Cell/Low‐Input RNA Library Prep Kit for Illumina (New England Biolabs, Ipswich, MA, US) according to the manufacturer's instructions. cDNA strand is first synthesized, which is amplified by PCR and then fragmented. Next, cDNA ends are repaired and adenylated. The NEBNext adaptor is ligated followed by second strand removal, uracil excision from the adaptor, and PCR amplification. Library sizes are checked using the Agilent 2100 Bioanalyzer, and the concentration is determined using the Qubit® fluorometer (Life Technologies). Libraries are sequenced at 650 pM on a P3 flow cell of the NextSeq 2000 (Illumina) to generate 60‐base single reads.

FastQ files for each sample are obtained using the bcl2fastq 2.20 Software (Illumina).

### Quality check of the reads and bioinformatic tools for circadian analysis

The Nextflow nf‐core/rnaseq v.3.2 pipeline [26] is used (a) to map the raw FastQ reads to the reference genome using star aligner [27], (b) to project the alignments onto the transcriptome, and (c) to perform the downstream transcript‐level quantification with Salmon [28]. Gene‐level summarization of read counts and transcript per million (TPM) abundances is done using the r package tximport [29].

The following sample attributes are considered to filter high‐quality samples: the number of reads mapped, the percentage of guanine‐cytosine (GC) content, the percentage of reads mapped to the mitochondrial genome, and the distance from other samples of the group/ZT (Zeitgeber time) in principal component analysis (PCA). Correction for batch effects is applied: (a) to read counts at the stage of linear modeling by r packages deseq2 [30] or by dryr for differential expression and differential rhythmicity analyses, or (b) directly to TPM using the ‘ComBat’ function of the sva r package [31] prior to identifying rhythmic genes in individual groups. Batch‐corrected TPM matrices are also used as an input to the gene set enrichment analysis (GSEA) [32].

DESeq2 can be used with default parameters and can include samples from all time points into the model, to define the lists of genes considered as differentially expressed between two groups, if the *P*‐adjusted threshold is < 0.05. To evaluate biological functions differentially affected regardless of their rhythmicity, gsea software is used with the following specific parameters: ‘gene_set’ permutation type and the ‘*T*‐test’ metric for ranking genes using the median for class metrics instead of the mean. An FDR *q*‐value threshold of 0.25 is used to delineate significant gene set enrichment.

For all the described enrichment analyses, we use Molecular Signatures Database (MsigDB) [33] Gene Ontology biological processes (GOBP) [34], canonical pathways (KEGG and Reactome) [35, 36], and custom‐designed gene sets.

Many algorithms are available for differential rhythmicity analysis of circadian datasets. Classically used methods in the field include JTK_CYCLE [24] (symmetric waveforms) and RAIN [12] (asymmetric waveforms). Caution must be taken when using such algorithms for comparing differential gene expression between groups, due to reported overestimation of differences [15]. In response, the field has developed algorithms more suitable for differential comparison such as limorhyde [18] (linear models for rhythmicity, design) and dryr [21]. We have still found the classic algorithmic approaches to be of descriptive use and biologically useful information may still be extracted by using an additional *q*‐value cutoff for the PSEA (such as described below for JTK_CYCLE in combination with PSEA). Using JTK_CYCLE, which provides phase and amplitude outputs for detected rhythmic genes with cosine expression waveform over a period of 24 h, rhythmic genes are first selected by the adoption of a threshold (See Tips below) such as adjusted *P*‐value < 0.05 as has been used previously for muscle stem cells [23]. To map rhythmic pathways and processes of muscle stem cells to the temporal scale, PSEA is performed on JTK_CYCLE‐identified genes with the following parameters: domain from 0 to 24 h, a minimum of 10 genes per gene set, maximum 1 million simulations, with a Kuiper *q*‐value <0.05. The enrichment is tested against a uniform background distribution to summarize any overall synchronization of peak phases within gene sets. To account for asymmetric waveforms, as an alternative method to call genes rhythmic in each genotype/condition, we choose RAIN, which can capture not only sinusoidal oscillations but also ‘sawtooth’ or ‘spiky’ patterns of gene expression [12]. As for JTK, caution must be taken during downstream analyses on gene sets identified using RAIN if comparing between conditions. Indeed, due to the multi‐group nature of our analyses, we now use the dryR R package to define rhythmic classes of genes; additional details on its use can be found at this link https://github.com/naef‐lab/dryR. For pairwise comparisons using dryr r package, it is also necessary to apply cut‐offs during data processing‐ here termed Bayesian information criterion weight (BICW). The choice of BICW that is appropriate to use changes according to the number of groups. For example, for two group analyses, BICW > 0.95 has been used [21] whereas BICW > 0.4 was used for four group comparisons. In our experience, we found a BICW > 0.6 to also be appropriate for four group comparisons. In accordance with the original publication, we use these BICW cut‐offs with the following: amplitude > 0.25 and Cook's distance < 1. Enrichment of MsigDB gene sets with rhythmic genes corresponding to gain, loss, phase change, and same rhythm categories are performed using a hypergeometric test, with significance defined by Benjamini–Hochberg (BH) adjusted *P*‐value < 0.05. Network representation and clustering of differentially rhythmic gene sets are performed based on common genes and semantic similarity using EnrichmentMap [37] and AutoAnnotate [38] plugins for Cytoscape [39]. References corresponding to the methodological tools discussed in this paragraph are summarized in Table 1.

**Table 1:** Methodological tools.

Technique	DOI
Lomb–Scargle periodograms	10.1093/bioinformatics/bti789
Harmonic regression analysis	10.1093/bioinformatics/btq189
JTK_CYCLE	10.1177/0748730410379711
RAIN	10.1177/0748730414553029
eJTK	10.1371/journal.pcbi.1004094
BIOCYCLE	10.1093/bioinformatics/btw243
PSEA	10.1177/0748730416631895
DODR	10.1093/bioinformatics/btw309
LimoRhyde	10.1177/0748730418813785
CircaCompare	10.1093/bioinformatics/btz730
Limma	10.1093/nar/gkv007
dryR	10.1073/pnas.2015803118
nf‐core framework for bioinformatics	10.1038/s41587‐020‐0439‐x
STAR RNA‐seq aligner	10.1093/bioinformatics/bts635
Salmon for transcript expression	10.1038/nmeth.4197
DESeq2 differential expression	10.1186/s13059‐014‐0550‐8
Batch removal with sva	10.1093/bioinformatics/bts034
GSEA	10.1073/pnas.0506580102
MSigDB	10.1093/bioinformatics/btr260
Enrichment map for GSEA	10.1371/journal.pone.0013984
AutoAnnotate for Cytoscape	10.12688/f1000research.9090.1
Cytoscape	10.1101/gr.1239303

## Tips & tricks/troubleshooting

### Quality control

#### Tip 1

Algorithms used to define the rhythmic transcriptome are often sensitive to outliers. Considering the high number of samples used in the circadian transcriptome analysis and the low amounts of RNA from muscle stem cells, the chance of having an outlier among the replicates of a single or a few time points is high. Furthermore, cell isolation and FACS sorting procedures could considerably affect the viability of cells, which may further skew the circadian transcriptome toward the markers of cell damage. Thus, it is necessary to include spare replicates and to perform extensive quality control of the sequenced samples prior to identifying rhythmic genes.

#### Tip 2

Correction for any technical (e.g., RNA extraction date) or biological (e.g., sex of the mice) batch effects is essential for decreasing the intra‐group variability of the gene expression peaks [40]. It is worth mentioning that a higher number of rhythmic genes could be detected in sexually homogeneous than in sexually heterogeneous experiments.

#### Tip 3

**Fig. 1:**
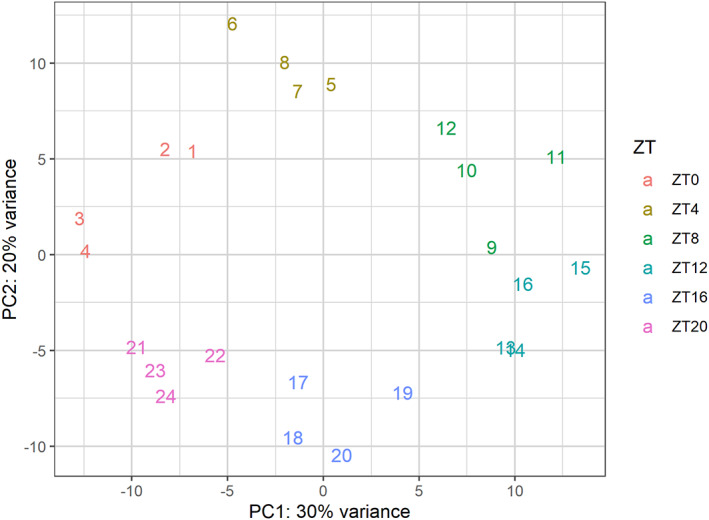
PCA plot of the rhythmic transcriptome resembling a clock face. Each color represents a separate time point, while the numbers correspond to replicate number.

At the end of quality control, cleaning, and batch correction procedures, the arrangement of high‐quality samples in the 2D PCA space might resemble a clock face (see Fig. 1).

### Rhythmic gene detection

#### Tip 1

**Fig. 2:**
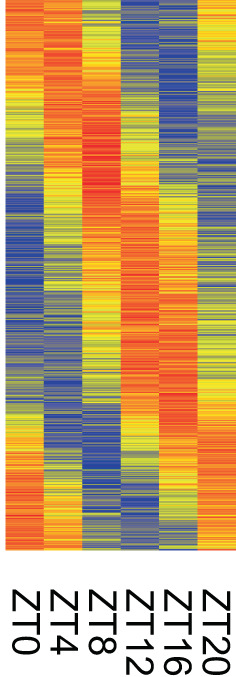
Heatmap of scaled and zero‐centered rhythmic gene expression at each time point. Genes are sorted by their rhythmic phase. Red corresponds to the maximum expression level and blue to the minimum.

There is no gold standard for the *P*‐value threshold in chronobiology due to intrinsic differences between the algorithms [24]. For example, the *P*‐adjusted threshold < 0.05 will be very permissive for BH‐corrected RAIN results and extremely conservative for filtering JTK results by ‘BH.Q’, or BioCycle results by ‘Q_VALUE’. In order to define a relevant *P*‐value threshold, we utilize the heatmaps of scaled and zero‐centered rhythmic gene expression (see Fig. 2) and visual inspection of the expression plots for individual genes.

#### Tip 2

**Fig. 3:**
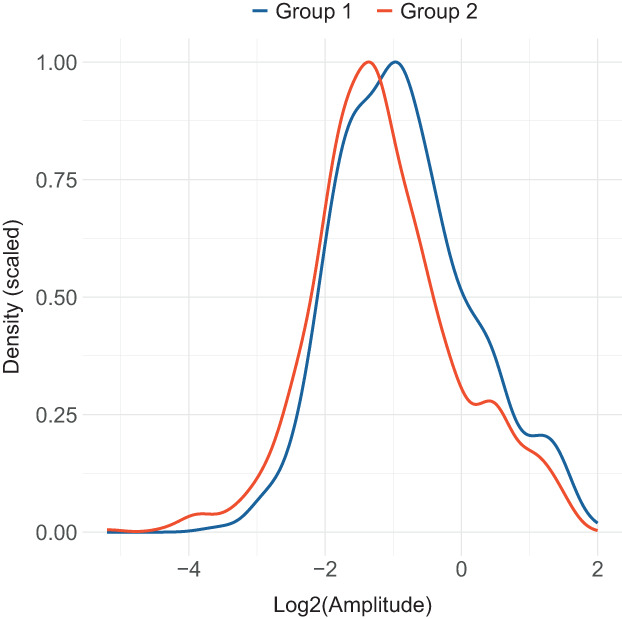
Scaled density of log2‐transformed amplitude distributions plotted for two experimental groups.

Amplitude size (Fig. 3) is also an important parameter when evaluating the differences in circadian rhythmicity between conditions. The density of log‐transformed amplitude distribution serves as a good means for visual comparison, while two‐sided *t*‐test statistics applied to the nontransformed amplitude distributions help to quantitatively estimate the difference in pairwise comparisons.

### PSEA

#### Tip 1

While high numbers of simulations are necessary to decrease the number of gene sets with *P*‐values equal to zero, setting this parameter to 1 million may result in an excessively long computational time proportional to the length of the input gene list. The maximum number of simulations can be decreased if the input files contain high numbers of rhythmic genes.

#### Tip 2

As an alternative to using all rhythmic genes as an input for PSEA, one may try taking the top N rhythmic genes ranked by significance for each ZT. This could be useful for flattening the distribution of rhythmic genes (by cutting down the biggest peak) to give PSEA less chance to center the phase of all pathways at the biggest peak, thus spreading out phase distribution more evenly. This may also help to standardize the number of gene sets enriched across the experiments if the same N is used. However, the pitfall is that this threshold is very arbitrary, and many relevant genes (and thus gene sets) may be missed.

#### Tip 3

**Fig. 4:**
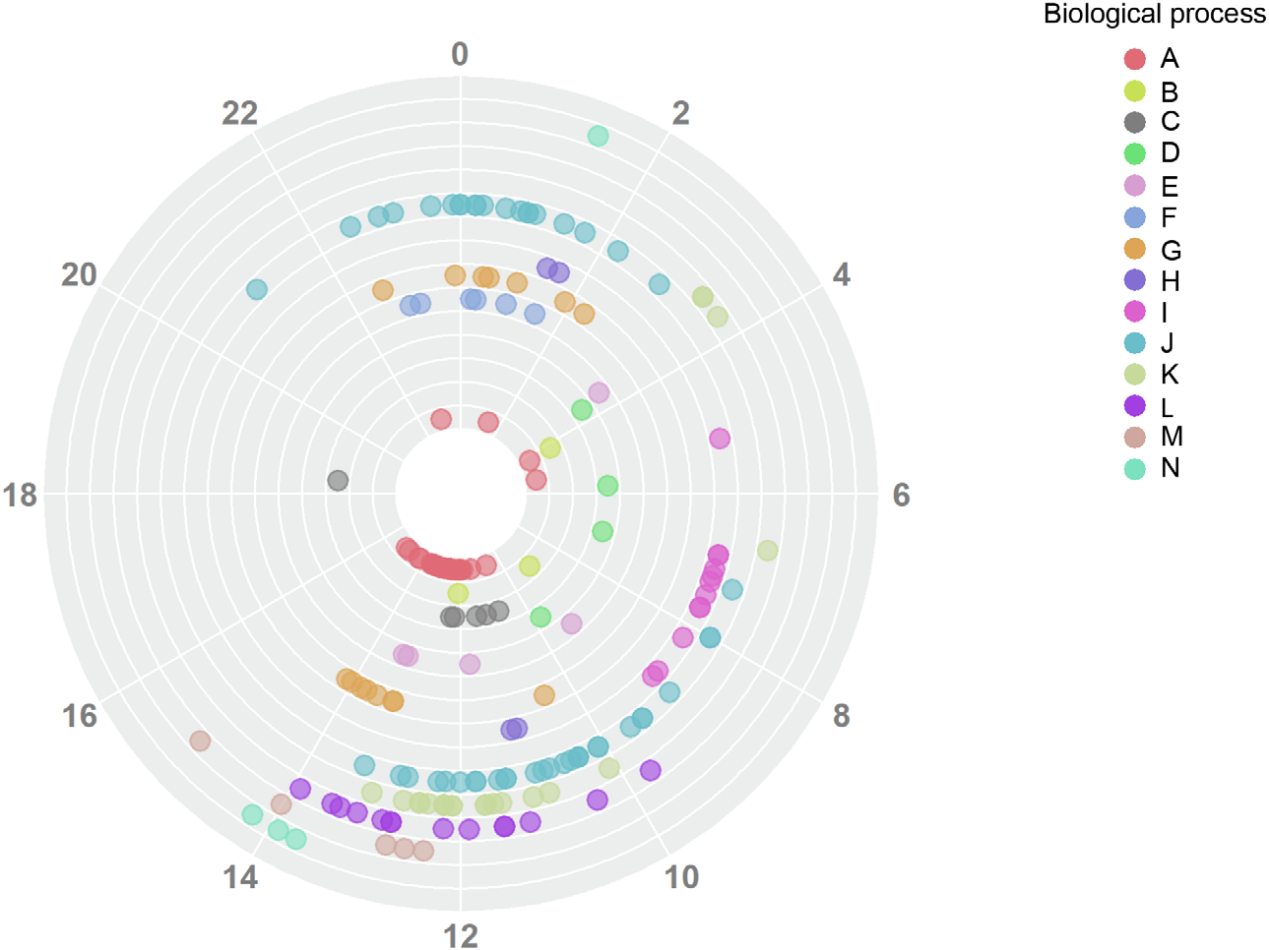
Example of the circular plot showing the distribution of gene set vector‐average values from the results of PSEA analysis. Each color corresponds to a separate functional cluster.

We are using a semi‐automated approach to aggregate individual gene sets into broad muscle stem cell‐oriented functional categories based on semantic similarity, positions in hierarchical trees of corresponding databases, and the analysis of gene set descriptions. This allows us to get distributions of peak phases (vector‐average values) within the main rhythmic functions and to visualize their span and temporal synchronization across the diurnal cycle using r package circlize [41] (see Fig. 4).

### DryR

#### Tip 1

An increase in the number of group sizes also can lead to an increase in the number of rhythmic categories for each newly‐included group; BICW values may have to be adjusted to account for this.

#### Tip 2

To facilitate the definition of the threshold for the BICW, we recommend plotting its distribution density for each comparison.

**Fig. 5:**
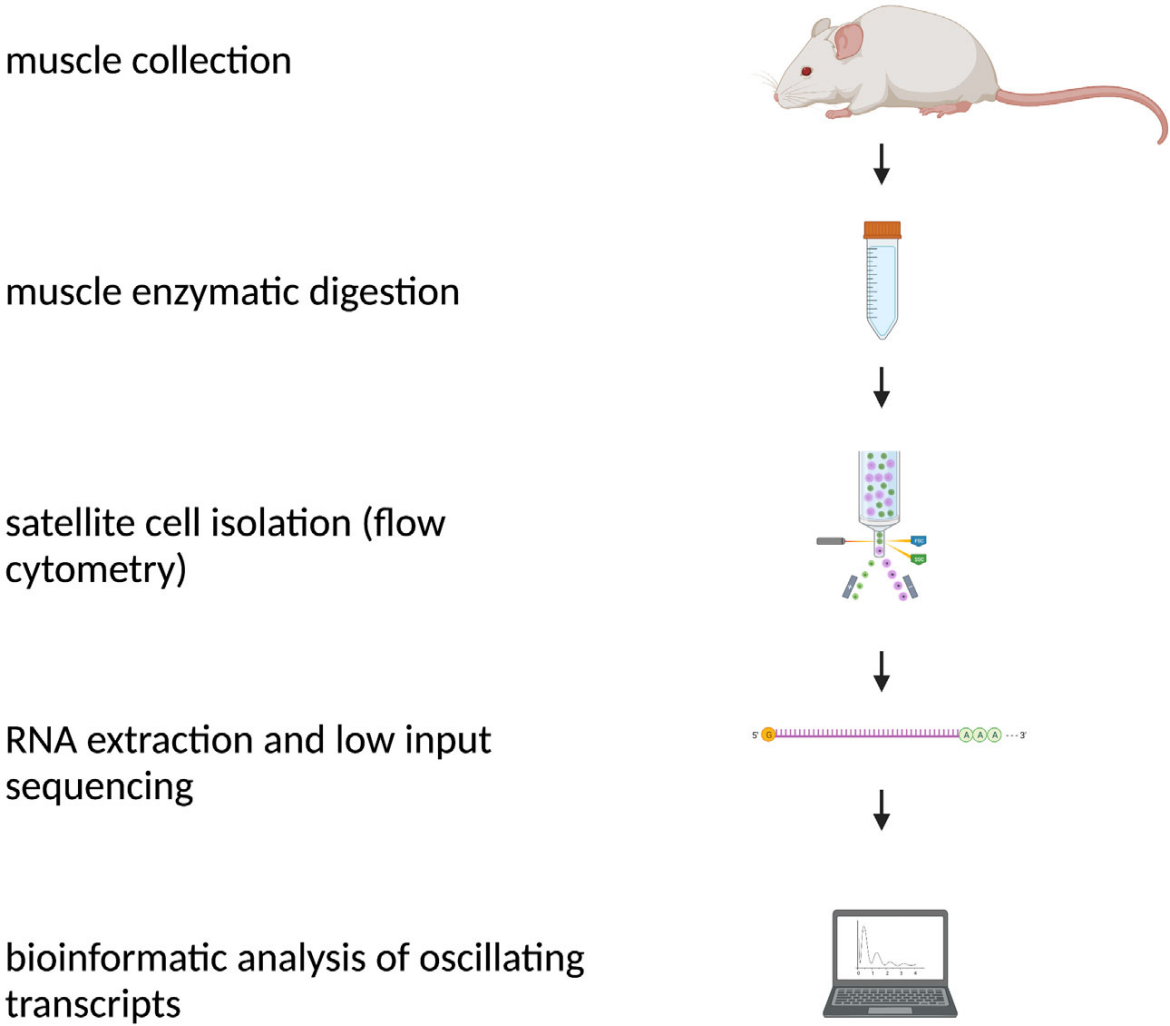
Schematic workflow.

A schematic workflow is provided in Fig. 5.

## Conflict of interest

The authors declare no conflict of interest.

## Author contributions

VS and OD wrote the manuscript, JGS edited the manuscript and wrote sections. PM‐C supervised and wrote the manuscript.

## Data Availability

The data included in the figures are only for demonstration purposes and are part of an ongoing investigation that is in the process of publication.
